# Effects of the Paediatric Regulation funding on the development of off-patent medicines in children

**DOI:** 10.3389/fmed.2024.1473862

**Published:** 2025-01-30

**Authors:** Lucia Ruggieri, Silvia Torretta, Viviana Giannuzzi, Alessandra Natale, Mariagrazia Felisi, Adriana Ceci, Fedele Bonifazi

**Affiliations:** ^1^Department of Research and Innovation, Fondazione per la Ricerca Farmacologica Gianni Benzi Onlus, Bari, Italy; ^2^TEDDY European Network of Excellence for Paediatric Research, Pavia, Italy

**Keywords:** paediatric medicines, off-patent, repurposing, Seventh Framework Programme, European Paediatric Regulation, public-private partnership

## Abstract

**Introduction:**

In paediatrics, medicines repurposing is a particularly advantageous approach, offering a route to address unmet medical needs and turn off-label use into evidence-based treatments for paediatric populations. This study analysed the effects of funds provided under the Seventh Framework Programme for Research (FP7-FRP), issued by the European Commission from 2007 to 2013 according to the European Paediatric Regulation, in terms of new paediatric marketing authorisations (MAs) including paediatric Use Marketing Authorisations (PUMAs). Additionally, we investigated which funded projects included repurposing initiatives.

**Methods:**

Data was collected on paediatric Investigation Plans (PIPs), new MAs, and MAs variations from the EMA website, national medicine registers, and final project reports. A survey to project coordinators was also conducted to explore the challenges faced during paediatric drug development plans.

**Results:**

The 20 FP7-funded projects studied 24 off-patent active substances. Eighteen substances had agreed PIPs with the European Medicines Agency paediatric Committee (PDCO). Positive compliance checks were granted for three PIPs, resulting in three new PUMAs. According to the adopted definition, 22 out of 24 (91.6%) paediatric development plans could be classified as repurposing. New conditions were proposed for eight substances, while 16 aimed to extend existing indications to broader paediatric populations. Additionally, 18 development plans included new age-appropriate formulations. The survey revealed that primary challenges in paediatric development plans included budgeting, lengthy regulatory processes, and recruitment.

**Discussion:**

Taken together, these results highlighted on one hand that the FP7 programme had a positive impact, as three new PUMAs were effectively obtained, representing one third of the nine PUMAs obtained since the paediatric Regulation entered into force, and three out of 18 agreed PIPs were successfully completed within 3–10 years. In addition, repurposing existing drugs for paediatric use significantly contributed to addressing unmet medical needs in paediatrics. On the other hand, the gap between the number of agreed PIPs and those that have led to PUMAs is still considerable, due to regulatory barriers and financial constraints. This underscores the need for continued support and further initiatives to streamline public-private partnerships for paediatric drug development, ensuring that off-patent medicines can be safely and effectively repurposed for paediatric use.

## 1 Introduction

Despite the absence of a regulatory definition, medicines repurposing (or repositioning) is intended as new therapeutic use for an existing medicine/active substance for an indication outside its existing authorised indication (1).^1^ Repurposing primarily involves bringing new therapeutics uses on label for already known medicines that are off-patent and no longer under regulatory data protection.^2^ Repurposing existing drugs for new uses is considered a more time- and cost-effective approach than developing new drugs, leading to higher success rates.

This approach is crucial in paediatrics, as it enables for the acknowledgment of new indications for existing drugs, potentially accelerating the availability of treatments for paediatric patients and cover unmet therapeutic needs (2, 3). Therefore, it can turn an off-label use of medications in children, which is widespread among paediatricians, into a safer alternative with appropriate dosing, efficacy and safety in specific paediatric populations (4, 5).

Thus, overall, repurposing paediatric medicines holds promise for addressing unmet medical needs in children and improving healthcare outcomes (2, 6). Several studies have highlighted the success of repurposing drugs for conditions such as paediatric cancers, hematological disorders, and rare genetic diseases (2, 3, 7, 8). Additionally, the use of real-world data in drug repurposing has recently emerged due to the recognised benefits of incorporating real-world evidence in developing medicines for small populations and for regulatory approvals (9).

The innovative uses of medication in paediatrics through repurposing were analysed in a systematic review published in 2016 by Rumore (3). The analysis showed that this approach mainly involved older generic or widely used medications with minimal evidence of harm to children, and yet few new medications were repurposed in paediatrics.

Although some non-experimental evidence on the benefits of off-label use may be available in clinical practice, off-label uses lack scientific validation for efficacy and safety (10). Research is paramount to establish safety and efficacy, as off-label use without therapeutic benefit can lead to wasted medication and potential patient risk (3).

With the aim to improve evidence-based treatment in small populations, including the paediatric population, academia, pharma companies, patients' associations, and other not-for-profit organisations are often involved in public-private partnerships to gather or generate sufficient evidence for a new indication of medicines with a well-established use.^3^

To date, off-patent medicines can be developed for new indications in both paediatrics and adults.^4^ This requires the submission of appropriate toxicological and pharmacological tests and/or of clinical trial data, without the need for a dedicated Paediatric Investigation Plan (PIP). As a reward, developers receive 1 additional year of marketing protection for medicines including a substance with well-established use with one or more new therapeutic indications and 1 year of data exclusivity for a new indication.

In 2007 the European Paediatric Regulation^5^ entered into force, including two groups of further provisions/incentives to support research on off-patent medicines:

1. A dedicated marketing authorisation (MA) covering the indication(s) and appropriate formulation(s) for medicines developed exclusively for use in the paediatric population, the *Paediatric-Use Marketing Authorisation* (PUMA, Article 30 of the European Paediatric Regulation). The development of a PUMA must follow a voluntary PIP, to be agreed by the Paediatric Committee (PDCO) within the European Medicines Agency (EMA). Once obtained, the PUMA grants holders the benefit of 8 plus 2 years of data and market protection, if studies are performed as per an agreed PIP.
2. *Funding for research to cover the development of off-patent medicinal products* (European Paediatric Regulation article 40, 1). In order to ensure that funds were directed to research of medicinal products with the highest needs in the paediatric population, the PDCO adopted a priority list of off-patent products for which studies are required.^6^ These funds were delivered through competitive calls from proposals under the Seventh Framework Programme for Research^7^ (FP7-FRP), issued by the European Commission from 2007 to 2013.

These provisions aimed to meet the specific needs of researchers and experts in paediatrics in Europe by supporting them in developing innovative study designs, preparing protocols for paediatric interventional clinical trials, and accessing funding opportunities (11).

The calls for projects funded under the FP7-FRP focused on innovative research methodologies and development in various health areas, aiming to develop new treatments, improve healthcare outcomes, and understand disease mechanisms. FP7-FRP utilised various funding schemes, including collaborative research projects and networks to connect researchers across disciplines and countries.

A preliminary analysis of achievements and issues encountered by projects receiving FP7-FRP funds for paediatric development of off-patent medicines was published in 2015 (12), when most of the projects and related studies were ongoing or just started. This analysis revealed a successful impact of the Paediatric Regulation and a substantial contribution to paediatric research. The 20 approved projects received 98.6 million euros, facilitating 71 paediatric studies including 29 paediatric clinical trials. Most projects focused on developing age-appropriate formulations to address unmet needs in paediatrics (12).

Nearly a decade later, this work aimed to analyse the effects of the funds provided under the Article 40 of the Paediatric Regulation in terms of new paediatric MAs, including PUMAs, particularly emphasizing if the funded projects were aimed at repurposing.

As secondary objectives, this work explored the challenges of conducting paediatric investigation plans of off-patent medicines in public-private consortia supported by European public funds.

## 2 Materials and methods

### 2.1 Study sample

For the purpose of this study, the list of off-patent active substances studied in an FP7-funded project (Area 4.2-1 Responding to EU policy needs: Off-Patent Medicines for Children) was taken into account, as previously reported (12). A total of 24 active substances were analysed covering 10 therapeutic areas in all paediatric age groups.

### 2.2 Study variables

#### 2.2.1 Outcomes of FP7 funding on paediatric medicines development

To analyse the outcomes of the FP7 funding on new MAs for paediatrics and/or variations to existing MAs, the following information was retrieved for each active substance:

- Details of any PIPs agreed by EMA-PDCO (Regulation (EC) No 1901/2006): PIP application number, indication(s), age groups, completion date, and compliance check.
- Details on any new MAs (including PUMAs) or variations to existing MAs obtained following the R&D funded by the FP7 programme: tradename, active substance, therapeutic areas, indication and age groups, routes of administration, pharmaceutical forms, and strengths.

To understand if projects funded under the FP7 programme were aimed at proposing a paediatric repurposing, the following information was retrieved for each active substance:

- Details on existing MAs at national/centralised level: approved indication, presence of a paediatric indication, authorised pharmaceutical forms.
- Therapeutic indication(s) and pharmaceutical formulation(s)/form(s) under development within FP7 projects.

In addition, it was verified if the therapeutic indication referred to a paediatric-only diseases. Information was retrieved and assessed by two different researchers in literature. Advice was also requested from an expert in the paediatric research field (AC).

#### 2.2.2 Data sources

- The European Medicines Agency (EMA) website^8^ for details on PIPs (including measures, timelines and number of modifications), European Public Assessment Reports of centrally authorised medicines, Orphan Designations (OD) and authorised medicines included in the EMA article 57 databases.^9^
- The final reports of the projects to confirm that the PIPs and the new MAs were referred to the specific FP7-funded project.^10^
- The 27 national medicine registers of the EU Member States, available on the EMA website,^11^ were initially consulted to retrieve MAs details of active substances authorised at the national level. After evaluating the completeness, updating frequency, and registry size in terms of both population coverage and number of medicines included, the Italian,^12^ French,^13^ and German^14^ national medicines registers were deemed suitable for our analyses, and subsequently consulted. The translation of information included in national registers to English has been performed using the DeepL translation system, in the freely available version (https://www.deepl.com/it/translator). Moreover, the free version of DeepL Write (https://www.deepl.com/it/write) was used to review grammar and style in certain sections of this manuscript.
- The EMA database of referral procedures,^15^ to identify “harmonisation” efforts aimed at resolving discrepancies in the authorisation of nationally authorised medicinal products.

Information was retrieved and assessed by two different researchers, and the data were cross-checked. A third researcher was involved to resolve any discrepancy.

#### 2.2.3 Survey on challenges of conducting paediatric development plans

To explore the challenges encountered during the conduct of these development programs, a survey (Supplementary material) was addressed to the scientific coordinators/project managers or their officially designated delegates, for a total of 21 recipients, investigating two main areas:

- Progression of the paediatric development plan, including the experience with the PIP agreed with EMA-PDCO and the obtainment of an EU MA.
- Main challenges experienced during the conduct of the project and the R&D.

Contact information was obtained through Community Research and Development Information Service (CORDIS) database.^16^

The survey was developed using Microsoft Forms™ (Microsoft Corporation, Redmond, WA, USA) and shared via email with recipients. Weekly reminders were sent, and follow-up emails were sent when clarifications were needed.

### 2.3 Data summary and analysis

Therapeutic indication(s) for each active substances retrieved by national and centralised MAs were compared to those developed within FP7 projects, including target population and age subsets. As no official definition of repurposing exists, repurposing was considered if a new therapeutic use for the active substances was envisaged (1). This also aligns with innovative pharma companies definition on repurposing (see text footnote 2).

Thus, the following cases were considered as repurposing:

- to extend its use to a *new non-authorised indication* (use for a different disease/condition than the authorised one);
- to extend its use to a *new paediatric age subset*;
- to develop a *new pharmaceutical age-appropriate formulation* through a new route of administration, treatment regimen or pharmaceutical form;
- to extend its use to a *wider therapeutic indication* covering more diseases due to the mechanism of action of the medicine,^17^ as well as to encompass other changes to the therapeutic indications, such as different stages or severity of a disease, shifting from first-line treatment to second-line treatment or vice versa, transitioning from combination therapy to monotherapy or from one combination therapy to another, and altering the approach from treatment to prevention or diagnosis of a disease or vice versa.

For the survey data, a descriptive analysis was conducted to summarise the collected information. This included indicating relative and absolute frequencies for each of the following categories:

- Budgeting.
- Long regulatory/ethics committee approval process for paediatric clinical trials.
- Recruitment challenges.
- Challenges with drug supply.
- Responding/complying with Paediatric Committee requests.
- Development of age-appropriate drug formulations.
- Safety/Efficacy issues.
- Responding/complying with national Competent Authorities and/or ethics committees requests.
- Trial sites activation.
- Coordinating different stakeholders/Withdrawal of key stakeholders.
- Conduct of non-clinical studies.

### 2.4 Ethical considerations

The study does not foresee the processing of personal data, except for the specific contacts details (i.e., full name and email address) of people interviewed for each project, which are publicly available on the CORDIS database. Personal identifiers were not disclosed, nor made available to third parties. Consent was obtained to respondents before completing the questionnaire to process the above-mentioned personal data, and only non-confidential aggregated information was reported.

### 2.5 Study period

Data collection of PIPs, MAs variations/PUMAs and consulting of national databases was performed from May 1st to June 30rd, 2024. All the MAs and PIPs granted until June 30rd 2024 were analysed. The survey for project coordinators/reference persons was conducted from May 16th to July 18th, 2024.

## 3 Results

### 3.1 Analysis of the outcomes of FP7 funding on paediatric medicines development

At the time of this analysis, all the 20 projects were officially concluded, in a time range spanning from August 2011 to July 2020. The projects studied 24 off-patent active substances of chemical origin. Among these, 18 (75%) active substances were studied according to a PIP agreed by the PDCO from November 2010 to November 2015, submitted in the framework of these projects (see Table 1).

**Table 1:** Details on agreed PIPs.

**Active substance**	**PIP application number**	**Paediatric indication**	**Age groups**	**Date of completion of the PIP**	**Compliance check**	**PUMA**
Azithromycin	EMEA-001298-PIP01-12	Prevention of bronchopulmonary dysplasia	From birth to <29 weeks of gestational age	Dec. 2017	No	NA
Budesonide	EMEA-001120-PIP01-10	Prevention of Bronchopulmonary Dysplasia in preterm newborn infants	From 23 to <28 weeks of gestational age (GA)	Jun. 2016	No	NA
Clonidine (hydrochloride)	EMEA-001316-PIP01-12	Sedation in intensive care	From birth to <18 years of age	Dec. 2017	No	NA
Cyclophosphamide	EMEA-000530-PIP02-11	Treatment of paediatric malignant diseases including hematological malignancies (including acute leukemia, malignant non-Hodgkin lymphoma, Hodgkin disease) as well as soft tissue sarcoma (including rhabdomyosarcoma, osteosarcoma and Ewing sarcoma), neuroblastoma and retinoblastoma	From birth to <18 years of age	Mar. 2015	No	NA
Deferiprone	EMEA-001126-PIP01-10	Treatment of iron overload in paediatric patients affected by hemoglobinopathies requiring chronic transfusion	From 1 month to <18 years of age	Sep. 2017	No	NA
Dobutamine (hydrochloride)	EMEA-001262-PIP01-12	Treatment of neonatal circulatory failure in the first 72 hours after birth	From birth to <1 month of age	Mar. 2023	No	NA
Dopamine	EMEA-001105-PIP01-10-M06	Treatment of hypotension in neonates including the extremely low gestational age newborn, in infants and children	From birth to <18 years of age	Apr. 2021	Granted on 14/10/2022	Granted on 27/05/2024 (Neoatricon^®^) for the treatment of hypotension in subjects aged 0–18 yrs
Enalapril (maleate)	EMEA-001706-PIP01-14-M02	Treatment of heart failure	From birth to <18 years of age	Apr. 2021	Granted on 25/06/2021	Granted on 15/11/2023 (Aqmeldi^®^) for the treatment of heart failure in subjects aged 0–18 yrs
Ethosuximide	EMEA-001617-PIP01-14	Treatment of childhood absence epilepsy	From 2 to <18 years of age	Jun. 2017	No	NA
Fentanyl (citrate)	EMEA-000712-PIP01-09	Prevention of acute pain, treatment of acute pain, pre-medication before a painful medical procedure	From birth to <2 years of age For pre-term neonates the corrected age according to maturity shall be taken into account	Jun. 2015	No	NA
Gabapentin	EMEA-001310-PIP01-12	Treatment of chronic pain of neuropathic origin	From 3 months to <18 years of age	Jun. 2020	No	NA
Hydrocortisone	EMEA-001283-PIP01-12	Replacement therapy in the treatment of adrenal insufficiency	From birth to <6 years of age	Oct. 2016	Granted on 14/10/2016	Granted on 09/02/2018 (Alkindi^®^) for the replacement therapy of adrenal insufficiency in infants, children and adolescents (from birth to <18 years old)
Meropenem	EMEA-000898-PIP01-10	Treatment of bacterial sepsis Treatment of bacterial meningitis	From birth to <3 months of age	Jun. 2015	No	NA
Metformin	EMEA-001352-PIP01-12	Treatment of polycystic ovary syndrome as adjunct to diet and exercise in adolescent girls to improve menstrual regularity and insulin resistance	Girls from 2 years post-menarche or from 14 years of age for patients with primary amenorrhea to <18 years of age	Nov. 2016	No, officially discontinued	NA
Morphine (hydrochloride)	EMEA-000711-PIP01-09	Treatment of moderate to severe prolonged pain.	From birth to <6 months of age	Jun. 2017	No	NA
Risperidone	EMEA-001034-PIP01-10	Treatment of conduct disorder in children and adolescents with average IQ	From 5 to <18 years of age	May 2015	No	NA
Temozolomide	EMEA-000530-PIP02-11	Treatment of malignant glioma, such as glioblastoma multiforme or anaplastic astrocytoma, with recurrence or progression after standard therapy and patients with difficulty swallowing	From 3 to <18 years of age	Apr. 2021	No	NA
Vancomycin	EMEA-001311-PIP01-12	Treatment of late onset bacterial sepsis caused by Vancomycin susceptible bacteria	From birth to <90 days of age	Mar. 2018	No	NA

The table includes information on the active substances studied, PIP application numbers, therapeutic indications, age groups covered as per the agreed PIP, completion dates, and compliance checks, where applicable.

For bumetanide, no PIP was finally agreed, due to the project consortium decision to divert attention to more promising development pathways, based on preliminary results obtained. For ciprofloxacin and fluconazole, the initial PIPs were not pursued further due to study modifications requested by the PDCO, that were deemed not feasible. The PIP for metformin was discontinued due to commercial reasons (Table 1).

Positive compliance check was granted from the PDCO for 3/18 PIPs (16.6% of agreed PIPs), including dopamine, enalapril, and hydrocortisone. Successful conclusion of these PIPs resulted in three new PUMAs granted at centralised level, hydrocortisone granules (Alkindi^®^), enalapril maleate (Aqumeldi^®^), and dopamine hydrochloride (Neoatricon^®^). Approval details on the PUMAs are available in Table 1.

A MAA (marketing authorisation application) for 6-mercaptopurine was withdrawn from the applicant as a similar product, having the Orphan Drug status, was granted a European MA in parallel and it was not possible to establish superiority.^18^ Further details on this specific case are reported in section “Challenges of conducting paediatric development plans.”

### 3.2 Repurposing associated to the development plans of the products

As off-patent medicinal products, all 24 active substances studied in the projects were approved at national level in at least one country considered in the analysis. Ten out of 24 active substances were also authorised at centralised level in EU. Six out of 24 (25%) active substances had an active OD at EU level for the same condition studied in the FP7 paediatric development plans. Three out of 24 (12.5%) development plans intended to study paediatric-only diseases, all including neonatal population. Details of the approved indications at centralised/national level are included in Supplementary Table 1.

According to the adopted definition, 22/24 (91.6%) paediatric development plans foreseen by FP7 projects could be classified as repurposing, as follows:

- New conditions, differing or wider from those approved for adults, were proposed for 8/22 (36.3%) paediatric development plans.
- For 16/22 (72.7%) cases, studies aimed to extend an existing indication approved for adults or other paediatric subsets to a broader paediatric population. In 18/22 (81.8%) plans, a new age-appropriate formulation/pharmaceutical strength was developed as part of these projects.
- Five out of 22 (22.7%) development plans included all the repurposing categories considered (Table 2).

**Table 2:** Repurposing of active substances for paediatric use.

**Active substance**	**Type of repurposing**
6-mercaptopurine^*^	New pharmaceutical age-appropriate formulation/strength
Azithromycin^*^	New non-authorised indication
	Extension to a new paediatric age subset
	New pharmaceutical age-appropriate formulation/strength
Budesonide	New non-authorised indication
	Extension to a new paediatric age subset
Bumetanide	New non-authorised indication
	Extension to a new paediatric age subset
	New pharmaceutical age-appropriate formulation/strength
Ciprofloxacin hydrochloride	Extension to a new paediatric age subset
	New pharmaceutical age-appropriate formulation/strength
Clonidine hydrochloride	New non-authorised indication
	Extension to a new paediatric age subset
	New pharmaceutical age-appropriate formulation/strength
Cyclophosphamide	Extension to a new paediatric age subset
	New pharmaceutical age-appropriate formulation/strength
Deferiprone^*^	Wider therapeutic indication
	Extension to a new paediatric age subset
	New pharmaceutical age-appropriate formulation/strength
Dobutamine (hydrochloride)	New non-authorised indication
	New pharmaceutical age-appropriate formulation/strength
Dopamine	Extension to a new paediatric age subset
	New pharmaceutical age-appropriate formulation/strength
Enalapril (maleate)	Extension to a new paediatric age subset
	New pharmaceutical age-appropriate formulation/strength
Ethosuximide	New pharmaceutical age-appropriate formulation/strength
Fentanyl (citrate)	Wider therapeutic indication
Fluconazole	Extension to a new paediatric age subset
Gabapentin	Extension to a new paediatric age subset
	New pharmaceutical age-appropriate formulation/strength
Hydrocortisone^*^	Extension to a new paediatric age subset
	New pharmaceutical age-appropriate formulation/strength
Meropenem	Extension to a new paediatric age subset
Metformin	New non-authorised indication
	Extension to a new paediatric age subset
	New pharmaceutical age-appropriate formulation/strength
Methotrexate	New pharmaceutical age-appropriate formulation/strength
Morphine	Extension to a new paediatric age subset
	New pharmaceutical age-appropriate formulation/strength
Temozolomide^*^	New pharmaceutical age-appropriate formulation/strength
Vancomycin	Extension to a new paediatric age subset
	New pharmaceutical age-appropriate formulation/strength

This table outlines the active substances studied in FP7 projects where repurposing was evaluated in comparison to the authorised indication, along with the type of repurposing according to the adopted definition.

^*^Active substance having an ODD for the same condition studied in the FP7 paediatric development plan.

Remarkably, more than one half (12 out of 22, 54.5%) of these repurposing development plans involved neonates, targeting diseases with no approved treatments and relying heavily on off-label use for management. Further details are available in Table 2 and in Supplementary Table 1.

### 3.3 Challenges of conducting paediatric development plans

Out of 20 inquiries in the survey, 19 (95%) coordinators/reference persons responded, indicating the status and the challenges of running paediatric development plans for off-patent medicines under the FP7 framework.

The survey results showed that 14/19 respondents (73.7%) declared their projects were concluded, 3/19 (15.8%) were still ongoing, while 2/19 (10.5%) did not start.

Among the concluded projects, 10/14 projects (71.4%) concluded the clinical phase, 3/14 (21.4%) projects stopped at the pharmaceutical development/manufacturing phase, and 1/14 (7.1%) was stopped at the registration phase. The latter refers to 6-mercaptopurine, whose MAA dossier for a new age-appropriate formulation was blocked at registration due to the concomitant registration of a similar product by a competitor.

Fifteen out of 19 (78.9%) respondents reported that a pharmaceutical company or SME, committed to apply for MA for the paediatric medicine, was involved in the consortium. However, details about their specific role and involvement in the projects were not provided. Financial difficulties faced by SMEs were also cited as challenges impacting the successful progress of these research programs.

**Figure 1:**
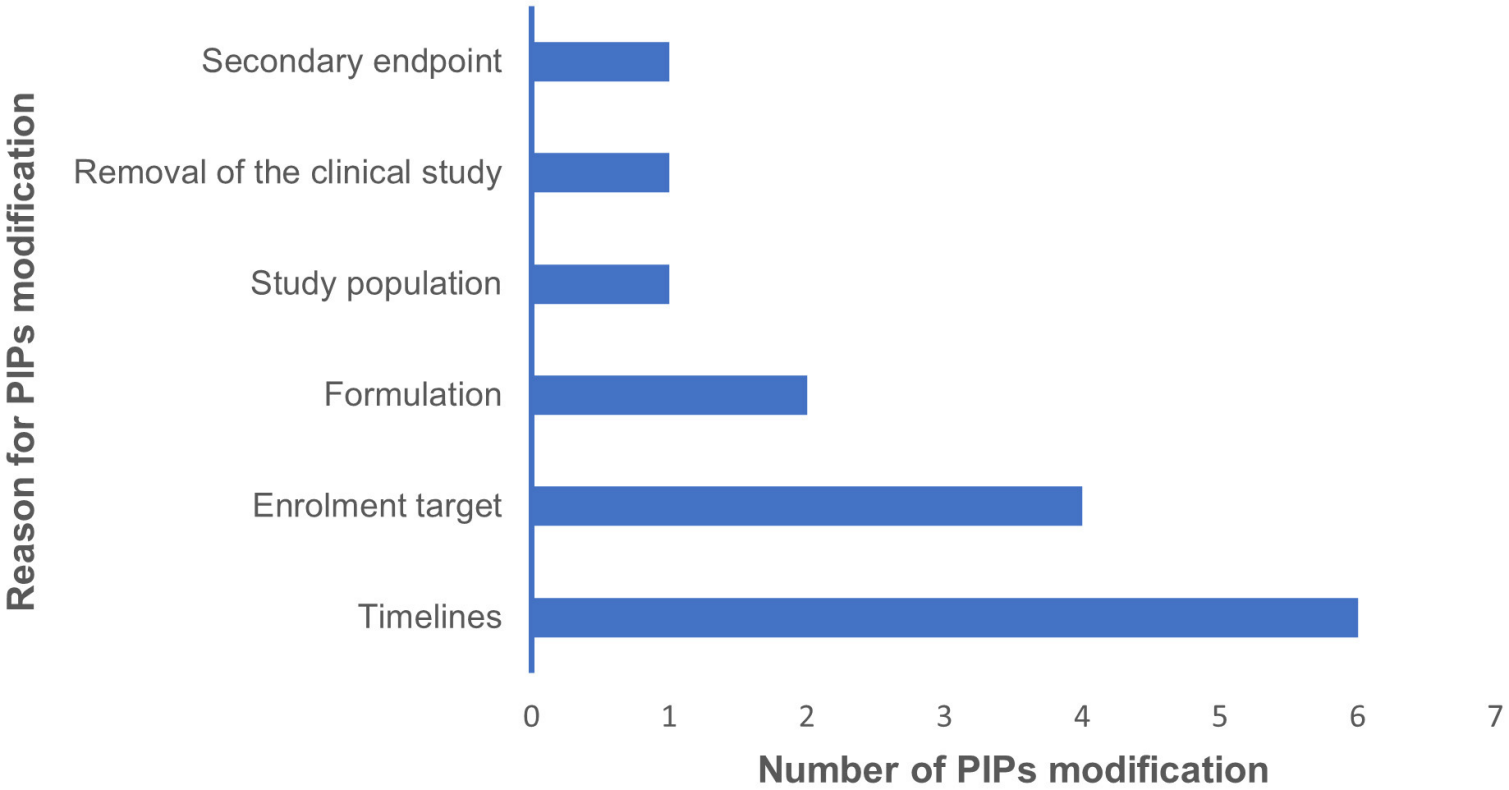
This bar graph summarises the main challenges in conducting a paediatric development plan, as identified in our survey, along with the number of respondents for each challenge.

In terms of challenges encountered during the paediatric development programs, the most frequently reported challenges were budgeting (8/19) and the long regulatory/ethics approval process for paediatric trials (8/19). Recruitment challenges were the next most common (7/19). Challenges with drug supply and complying with PDCO requests were cited by 6/19 respondents. The development of age-appropriate drug formulations was identified by 5/19 respondents. Safety and efficacy issues, along with complying with national Competent Authorities (CAs) and/or Ethics Committees (ECs) requests, were mentioned by 4/19 respondents each. Trial sites activation was noted by 3/19 respondents, while project partners coordination and the conduct of non-clinical studies were reported by 2/19 and 1/19 respondent(s), respectively (Figure 1). Purely scientific issues were not reported. Further explanations were provided about the following items:

a. Budgeting, timelines and logistics: resources were judged insufficient to meet the extended timelines and complexity required by these projects.
b. Poor attractiveness of the PUMA made unfeasible to have sponsors applying for a MA: the incentives of the PUMA were judged insufficient to reward the development costs and challenges involved. Sponsors argued that even if a PUMA was granted, competition would remain intense, due to the existence of competing generic products.
c. The costs associated with developing new age-appropriate products and conduct all the studies agreed at the EMA/PDCO level, were not duly acknowledged by national authorities responsible for pricing and reimbursement procedures, thus making the full development plans not sustainable.
d. Heterogeneous and burdensome regulatory procedures for clinical trial applications (CTA), especially in case of multi-center, multinational clinical trials requiring approvals from multiple ECs and CAs, substantially impacted on the success of these plans. The high number of modifications requested by various ECs/CAs led to the revision of protocols and related documents, generating in turn the need for substantial amendments submission.
e. Involvement of different countries also led to heterogeneity in handling clinical practice and professional roles/responsibilities and country-specific recruitment issues. Poor application of standardised regulatory requirements for the study management equivalent to the Good Clinical Practice (GCP) ICH E6(R3)^19^ guidance in extra-EU countries also negatively contributed to an effective setup of the clinical trial infrastructure.
f. Requirements posed at the EMA/PDCO level were highly impacting on the development plans conduct. These included, among the others, additional pre-clinical evidence on animal models asked through scientific advice procedures, changes to the clinical trial protocols in terms of study population, exclusion criteria, and comparator.
g. Ethical and cultural aspects in the informed consent process: collecting informed consent and defining the contents and format of information material for trial participants and parents/legal representative(s) was quite complex in multi-cultural scenarios.
h. Compliance with the requirements for the pharmaceutical development and manufacturing issues, technical aspects referring to the production of new formulations. Specific aspects were mentioned, such as humidity levels to be kept, drug component instability and complex chemical preparation, as well as failure to comply with GMP (Good Manufacturing Practice) requirements.
i. The occurrence of safety concerns requiring early termination of the clinical study (e.g., ototoxicity for one of the medicinal products).

**Figure 2:**
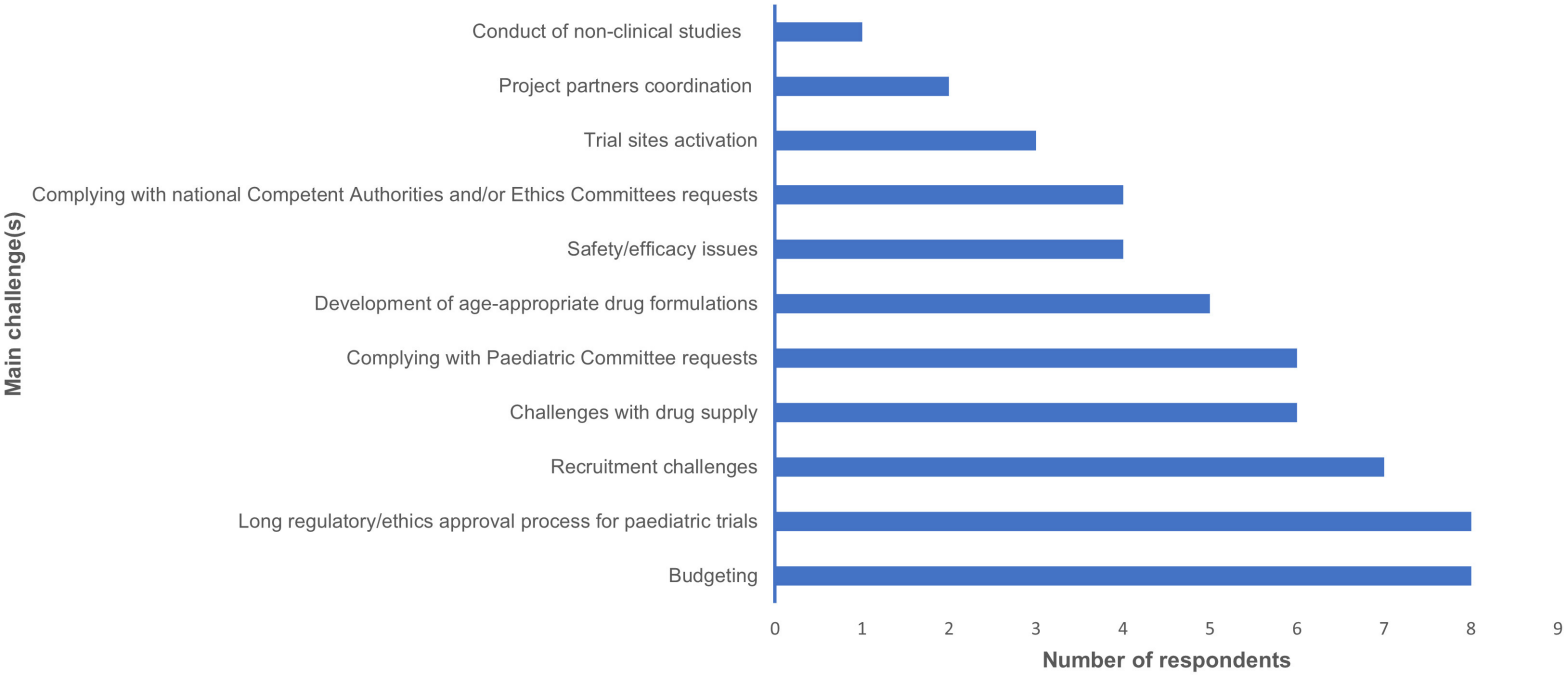
This bar graph summarises the main reasons why the respondents of our survey requested PIP modifications, along with the number of requests for modifications.

These challenges affected the conduct of studies included in PIPs agreed at the EMA/PDCO level. Indeed, 7/19 (36.8%) respondents declared they needed to ask for modifications to agreed PIPs. The main reasons for PIPs modification were mainly identified as difficulties in adhering to the scheduled timelines and challenges in reaching the enrolment target (Figure 2).

## 4 Discussion

To our knowledge, this is the only analysis of the outcomes from FP7 funding program aimed at achieving a PUMA. No other systematic investigation has been conducted to understand the effectiveness of EU investments in this area so far. Notwithstanding other networks in the field of paediatric clinical research have been funded through EU funding programmes (TEDDY,^20^ GRiP,^21^ PedCRIN,^22^ and c4c^23^), the FP7 one has some unique features. Firstly, it represents the only direct support for the implementation of specific paediatric drug development plans. Secondly, it directly results from applying the EU Paediatric Regulation provisions. In addition, it is one of the research programmes that succeeded in fostering the repurposing of off-patent products, even though repurposing was not its primary objective. Lastly, the FP7 programme enabled non-profit organisations to take an active role, even in partnerships with for-profit companies.

### 4.1 Outcomes of FP7 funding on paediatric medicines development

In terms of net results, three out of 20 projects succeeded in obtaining a PUMA for new paediatric medicines at centralised level. Although these results may be perceived as limited, representing a success rate of 15%, the following aspects need to be considered. First, two out of these three PUMAs were obtained in the last 2 years, more than 5 years after the formal completion of the project. So, other successfully completed projects could result in additional PUMAs in the future. Secondly, since the entry into force of the Paediatric Regulation, only nine PUMAs have been granted at EU level,^24^ thus those three resulting from FP7 projects represent one third of the total.

Most of the projects agreed a PIP with the EMA-PDCO and the PIPs completion rate was slightly higher than the PIP completion percentage as a whole [16.6 vs. 12% (13)].

Moreover, although most projects did not fulfill the primary objective of obtaining a PUMA, other outcomes were achieved. These included successful completion of paediatric clinical trials, development of new patents and technologies, dissemination of results through scientific publications, updating of clinical guidelines, conducting EU-wide surveys on paediatric clinical practice and updating state-of-the-art in niche therapeutic subsets, such as neonatal-only diseases and paediatric pain, as evidenced by the analysis of FP7 project reports. Remarkably, despite all the challenges in implementing these projects and plans, results from our survey emphasised that, without this funding, obtaining a centralised marketing authorisation would not have been possible.

### 4.2 Repurposing associated to the development plans of the products

Despite the projects were not specifically aimed at performing drug repurposing, they offered a good insight on the main challenges encountered by stakeholders engaged in paediatric drug development plans for old medicines being repurposed for in-label use. Most of the plans were intended to extend the use of the active substances beyond the current use, including adding new indications not approved in adults, extending existing indications to paediatric subjects and developing new age-appropriate formulations or pharmaceutical forms. For one third of active substances in the sample, the indication studied in the FP7 project was completely new from the ones approved in adults. For example, metformin is currently approved Europe-wide for type-2 diabetes treatment, whereas the reference FP7 project intended to generate pharmacokinetics, efficacy and safety data on its use for polycystic ovary syndrome (PCOS) in children (14), thus bringing this indication within its approved label. Remarkably, the PIP agreed by PDCO for metformin study in PCOS was discontinued due to commercial reasons, as the sponsor deemed the commercial opportunity in Europe insufficient to justify the development efforts.^25^ Similarly, the CloSed project intended to study clonidine for sedation in Paediatric Intensive Care Units generating evidence on its efficacy and safety, thus addressing an important paediatric therapeutic need. Unfortunately, the CloSed project did not complete its target due to financial difficulties of the sponsor, strict inclusion/exclusion criteria impacting on trial recruitment and long timelines for study approvals by ECs/CAs. In addition, within the DEEP project (15), the paediatric plan was successfully completed providing results supporting the extension of the use of deferiprone from thalassaemia major patients to children of all ages affected also by other rare hemoglobinopathies requiring chronic transfusions, such as the sickle cell disease. However, with only one recently authorised medicinal product in the EU^26^ for children aged more than 12, there is still an unmet medical need for rare hemoglobinopathies in paediatrics.

### 4.3 Challenges of conducting paediatric development plans

Some of the aspects mentioned by the respondents to the survey were common to all paediatric drug development plans: first of all, the regulatory issues related to multiple interactions with the ECs/CAs, as largely reported (16–19), which have a significant impact on paediatric clinical research (20). This is often due to a jeopardised expertise on paediatric matters at the ECs/CAs level (16, 21, 22).

Also, recruitment challenges reported by the coordinators are quite well-known in the paediatric clinical research landscape (23, 24), affecting different therapeutic settings (25, 26) and representing one of the most common reasons for early trials discontinuation in paediatrics (27, 28). Another specific challenge mentioned by the respondents is to comply with requests received by EMA-PDCO, leading to the withdrawal of several PIPs before their agreement. Although the impulse that the Paediatric Regulation gave to paediatric clinical research in the European setting (29), a series of shortcomings of the current regulatory framework are highlighted in literature: clinical trials are insufficiently adapted to the paediatric clinical practice and start too late compared to adult trials (30), leading to an average delay of 8 years in the availability of paediatric medicines (16, 31), the methodology adopted in some PIPs is a transposition of the adult development plan (32), the enrolment rate does not reflect the number of paediatric patients potentially eligible (30); the complexity and number of proposed studies do not reflect the reality of paediatric clinical practice (33). Last but not least, until now the system has not promoted the development of new drugs for neonatal/paediatric indications only (34, 35). These considerations led to a substantial reform of the European regulatory and legislative framework started in 2022 and involving the Paediatric Regulation.^27^ In fact, one of the new provisions introduced in this reform requires that medicinal products targeting a specific molecular pathway or possessing a mechanism of action linked to a different disease in adults (within the same therapeutic area) must also be studied in children.

However, some of the challenges we reported strictly reflect the poor attractiveness of repurposing off-patent medicines through the PUMA scheme,^28^^,^^29^ despite its original intent to be a key element of the Paediatric Regulation. In fact, the number and type of studies required in a PIP for a PUMA, the populations to be recruited, the complexity of the administrative/technical procedures, are similar to those required for new/innovative paediatric products,^30^ but running paediatric trials with off-patent medicines is affected by a higher degree of complexity (36). Consequently, developers prefer to apply under the simplified procedure outlined in the Directive 2001/83/EC for off-patent drugs rather than following the Paediatric Regulation route and discussing a PIP with the EMA-PDCO (35). Other common challenges reported by respondents and depending on the poor effectiveness of PUMA incentives refer to: the complexity of obtaining a modification to a product license, whether through a PUMA or otherwise, especially for non-commercial academic consortia; the economic value of medicines approved through a PUMA, as national health authorities responsible for pricing and reimbursement may limit revenues; and the small market size, due to the limited patient populations and the co-existence of generic competitors (13, 37, 38).

Specific attention must be paid to adequately support the efforts associated with the pharmaceutical development and manufacturing of new age-appropriate medicines formulations/strengths for old medicines. This is one of the main specificities of paediatric drug development and it has been cited as the stopping point for 3 FP7-funded projects. Indeed, the availability of cheaper options using extemporaneous galenic preparations might discourage developers from investing in new age-appropriate manufactured medicinal products, despite the challenges associated with extemporaneous preparations (39, 40). Notably, one of the primary reasons for trial failure in paediatric drug development is the absence of appropriate drug delivery systems (41). Medication palatability, swallowability and formulation are key elements of therapeutic drug-adherence and successful therapeutic outcome in paediatrics (42). Challenges in creating child-friendly formulations encompass clinical and technological aspects, including patient acceptance, the design of dosage forms, the selection of excipients, and the effects of dosage forms on pharmacokinetics and pharmacodynamics. Additionally, regulatory considerations, such as the limited size of the study population, as well as economic factors, and sustainability also play critical roles in the development process (42).

### 4.4 How to properly address the outcomes deriving from this analysis?

Based on these results, we can envisage that, despite research on novel medicines have an undoubtful relevance in addressing paediatric therapeutic needs, off-patent medicines being repurposed can still offer relevant treatment opportunities, also for rare neonatal diseases.

Thus, a series of support actions covering methodological and scientific aspects, as well as regulatory and legislative measures can make this process more effective. In this sense, several noteworthy initiatives on repurposing have been developed at EU and international level. The International Rare Diseases Research Consortium (IRDiRC)^31^ designed a guidebook to assist developers, including big pharma, clinicians, and patient-led initiatives, in repurposing existing drugs for new rare disease indications. A pilot call, jointly launched by Head of Medicines Agency/EMA in October 2021, provided free scientific advice at EMA/national level for not-profit-organisations and academia promoting repurposing development programmes.^32^ The aim of this initiative was to support, gather or generate sufficient evidence on the use of an established medicine in a new indication with the view to have this new use formally authorised by a regulatory authority. To date, several EU-funded networks are active on this topic. They include: REPO4EU,^33^ that brings together 28 partners from 10 countries to find new uses for existing drugs, reducing development costs and time, and advancing personalised therapies;, REMEDi4ALL,^34^ that is aimed at promoting patient-centric drug repurposing for rare and ultra-rare diseases, as well as other therapeutic areas; SIMPATHIC,^35^ focused on accelerating drug repurposing for rare neurological, neurometabolic and neuromuscular disorders by exploiting similarities in clinical and molecular pathology.

Although their work and contribution are commendable, no dedicated focus for paediatric repurposing has been found (as an example, none of them address the issue of developing age-appropriate formulations).

On the other side, paediatric drug development remains one of the main focus areas of clinical research in Europe, also thanks to the intense work that collaborative initiatives are performing with an intense resources' investments. Some relevant examples include conect4children, that aims to generate a sustainable infrastructure that optimises the delivery of clinical trials in children; TEDDY European Network of Excellence for Paediatric Research and EPTRI^36^ that are actively continuing their activities after the end of the funding period. Also, the paediatric clinical research consortia funded under the FP7-FRP represent a good example of public-private collaboration building their focus under a specific drug development plan and extending their activities also at the end of funding period. Last but not least, almost 40 paediatric specialty/multi-specialty, national/international networks are gathered under the umbrella of the European Network of Paediatric Research at the European Medicines Agency (Enpr-EMA)^37^ to support a broad range of activities, with the final aim of enhancing the development and availability of medicines for children across Europe.

Beyond the EU setting, at Food and Drugs Administration (FDA) level, the CURE-ID platform^38^ was launched in 2019 for health care professionals to report novel uses of existing medicines. Initially, it was focused on difficult-to-treat infectious diseases (including COVID-19), but now it includes information also on rare cancers and rare genetic disorders.

As support action, it would be useful to have collaborations between existing paediatric and paediatric initiatives to establish specific pathways for paediatric drug repurposing, starting from a common and shared definition.

Additionally, another improvement in the effectiveness of off-patent paediatric drug development plans can come from the revision of the current EU pharmaceutical legislation, including the Paediatric Regulation. Despite the wide debate around the PUMA concept and incentives, they remained unchanged in the current proposed text of the new legislation, that is expected to be finalised by 2027 (1).^39^^,^^40^ Thus, we do not expect any major impact on the number of PUMAs at EU level, also considering that the FP7 funding scheme has not been renewed as systematic support action, nor similar support actions are foreseen in the current legislation. Nevertheless, some positive effects can derive from the introduction of a more agile PIP system, the so called “initial PIP,” as many off-patent products currently registered in EU according to other regulatory tools (e.g., the Directive 2001/83/EC pathway) may be moved to PUMA if the PIP is simplified.

### 4.5 Strengths and limitations of this study

To our knowledge, this is the only in-depth analysis of the results of the FP7 funding to develop off-patent medicines, as derived from the obligation posed by the Paediatric Regulation. The qualified profile of respondents, as highlighted by their direct involvement in the projects, together with the high response rate, increases the value of the presented data. On the other hand, we acknowledge as a limitation that this work only analyses the effects of the funding scheme. Further insight into off-patent medicines developed according to the provisions of the Paediatric Regulation would be useful and could be pursued in the future.

## 5 Conclusions

This study shows that the FP7 programme has been somewhat successful: out of 20 projects on 24 active substances, 18 PIPs were agreed, three new PUMAs were effectively obtained following successful PIP completion, and 22 repurposing plans were developed to address unmet medical needs in paediatrics. However, it remains a gap between the number of agreed PIPs and those that have led to obtaining PUMAs, suggesting that further intervention is needed. Challenges such as regulatory barriers and financial constraints limited the success rates of this initiative and underline the need for continued support and streamlined processes. Public-private partnerships remain critical to advancing paediatric drug development and ensuring that off-patent medicines can be safely and effectively repurposed for paediatric use. Legislative initiatives and collaborative research networks funded at Eu level, including the Horizon Europe research and innovation funding programme,^41^ could also help to promote the use of drug repurposing for unmet medical needs in paediatrics.

## Data Availability

The raw data supporting the conclusions of this article will be made available by the authors, without undue reservation.

## Footnotes

^1^Question and Answers on repurposing pilot project on proposal for framework to support not-for-profit organisations and academia in repurposing authorised medicines. https://www.ema.europa.eu/en/documents/other/question-and-answers-repurposing-pilot-project-proposal-framework-support-not-profit-organisations-and-academia-repurposing-authorised-medicines_en.pdf. Accessed 8 July 2024.

^2^ https://www.efpia.eu/media/2mlhtlac/position-paper-on-drug-repurposing.pdf

^3^When an active ingredient of a medicine has been used for more than 10 years and its efficacy and safety have been well-established (EMA Glossary—Regulatory terms).

^4^Directive 2001/83/EC of The European Parliament and of the Council of 6 November 2001 on the Community code relating to medicinal products for human use. https://eur-lex.europa.eu/legal-content/EN/TXT/PDF/?uri=CELEX:32001L0083. Accessed 9 July 2024.

^5^Regulation (EC) No 1901/2006 of the European Parliament and of the Council of 12 December 2006 on medicinal products for paediatric use and amending Regulation (EEC) No 1768/92, Directive 2001/20/EC, Directive 2001/83/EC and Regulation (EC) No 726/2004 (Text with EEA relevance). https://eur-lex.europa.eu/legal-content/EN/TXT/?uri=celex%3A32006R1901. Accessed 8 July 2024.

^6^European Medicines Agency Funding for paediatric studies. https://www.ema.europa.eu/en/human-regulatory-overview/research-development/paediatric-medicines-research-development/funding-paediatric-studies. Accessed 20 November 2024.

^7^https://cordis.europa.eu/programme/id/FP7/it. Accessed 9 July 2024.

^8^https://www.ema.europa.eu/en/homepage. Accessed 22 July 2024.

^9^https://www.ema.europa.eu/en/human-regulatory-overview/post-authorisation/data-medicines-iso-idmp-standards-post-authorisation/public-data-article-57-database. Accessed 24 July 2024.

^10^https://cordis.europa.eu/search?q=contenttype%3D%27project%27%20AND%20frameworkProgramme%3D%27FP7%27&p=1&num=10&srt=/project/contentUpdateDate:decreasing&archived=true. Accessed 25 July 24.

^11^https://www.ema.europa.eu/en/medicines/national-registers-authorised-medicines. Accessed 22 July 2024.

^12^https://medicinali.aifa.gov.it/it/#/it/. Accessed 11 July 2024.

^13^http://agence-prd.ansm.sante.fr/php/ecodex/index.php. Accessed 11 July 2024.

^14^https://www.pharmnet-bund.de/PharmNet/DE/Oeffentlichkeit/Arzneimittel-Informationssystem/_node.html. Accessed 11 July 2024.

^15^https://www.ema.europa.eu/en/medicines/download-medicine-data#referrals-38551. Accessed 24 July 2024.

^16^http://cordis.europa.eu/home_en.html. Accessed 24 July 2024.

^17^https://health.ec.europa.eu/system/files/2016-11/10%252520_5_%252520guideline_11-2007_en_0.pdf. Accessed 24 July 2024.

^18^https://www.ema.europa.eu/en/documents/medicine-qa/questions-and-answers-withdrawal-marketing-authorisation-application-loulla_en.pdf, Accessed 25 June 2024.

^19^ICH Harmonised Guideline Good Clinical Practice (GCP) E6(R3). 19 May 2023. https://database.ich.org/sites/default/files/ICH_E6%28R3%29_DraftGuideline_2023_0519.pdf. Accessed 27 November 2024.

^20^Teddy European Network of Excellence for Paediatric Research. https://www.teddynetwork.net/. Accessed 26 July 2024. https://www.teddynetwork.net/. Accessed 26 July 2024

^21^Global Research in Paediatrics. https://cordis.europa.eu/project/id/261060. Accessed 26 July 2024.

^22^Paediatric Clinical Research Infrastructure Network. https://cordis.europa.eu/project/id/731046/it. Accessed 26 July 2024.

^23^Conect4children. https://conect4children.org/. Accessed 26 July 2024.

^24^Information retrieved through the EMA Ask service.

^25^https://cordis.europa.eu/project/id/602243/reporting. Accessed 27 November 24.

^26^https://www.ema.europa.eu/en/medicines/human/EPAR/casgevy. Accessed 27 November 2024.

^27^https://www.europarl.europa.eu/news/en/press-room/20240318IPR19419/eu-pharmaceutical-policy-meps-support-comprehensive-reform. Accessed 27 November 2024.

^28^European Medicines Agency (2017). 10-year report to the European Commission. General report on the experience acquired as a result of the application of the Paediatric regulation. EMA/231225/2015. https://health.ec.europa.eu/system/files/2020-06/paediatrics_10_years_ema_technical_report_0.pdf. Accessed 27 November 2024.

^29^European Commission (2020). Commission staff working document evaluation joint evaluation of regulation (EC) no 1901/2006 of the European Parliament and of the Council of 12 December 2006 on medicinal products for paediatric use and regulation (EC) no 141/2000 of the European Parliament and of the Council of 16 December 1999 on orphan medicinal products PART ⅙ (SWD(2020) 163 final). https://eur-lex.europa.eu/legal-content/EN/TXT/?uri=CELEX%3A52020SC0163. Accessed 27 November 2024.

^30^SWD (2023) 192 final. https://eur-lex.europa.eu/resource.html?uri=cellar:d75b91b3-e437-11ed-a05c-01aa75ed71a1.0001.02/DOC_2&format=PDF. Accessed 25 July 2024.

^31^IRDiRC - Drug Repurposing Guidebook. https://irdirc.org/drug-repurposing-guidebook/. Accessed 29 July 2024.

^32^https://www.ema.europa.eu/en/news/repurposing-authorised-medicines-pilot-support-not-profit-organisations-and-academia. Accessed 29 July 2024.

^33^https://repo4.eu/. Accessed 29 July 2024.

^34^https://remedi4all.org/. Accessed 29 July 2024.

^35^https://eatris.eu/projects/simpathic-accelerating-drug-repurposing-for-rare-neurological-neurometabolic-and-neuromuscular-disorders-by-exploiting-similarities-in-clinical-and-molecular-pathology/. Accessed 28 November 2024.

^36^European Paediatric Translational Research Infrastructure. https://eptri.eu/. Accessed 30 July 2024.

^37^https://www.ema.europa.eu/en/partners-networks/networks/european-network-paediatric-research-european-medicines-agency-enpr-ema. Accessed 29 July 2024.

^38^https://cure.ncats.io/home. Accessed 30 July 2024.

^39^Articles 92-93 Proposal for a REGULATION OF THE EUROPEAN PARLIAMENT AND OF THE COUNCIL laying down Union procedures for the authorisation and supervision of medicinal products for human use and establishing rules governing the European Medicines Agency, amending Regulation (EC) No 1394/2007 and Regulation (EU) No 536/2014 and repealing Regulation (EC) No 726/2004, Regulation (EC) No 141/2000 and Regulation (EC) No 1901/2006.

^40^European Parliament legislative resolution of 10 April 2024 on the proposal for a regulation of the European Parliament and of the Council laying down Union procedures for the authorisation and supervision of medicinal products for human use and establishing rules governing the European Medicines Agency, amending Regulation (EC) No 1394/2007 and Regulation (EU) No 536/2014 and repealing Regulation (EC) No 726/2004, Regulation (EC) No 141/2000 and Regulation (EC) No 1901/2006.

^41^https://research-and-innovation.ec.europa.eu/funding/funding-opportunities/funding-programmes-and-open-calls/horizon-europe_en. Accessed 27 November 2024.
