# Supplementary material for: Effects of the Paediatric Regulation funding on the development of off-patent medicines in children
Source: Front Med (Lausanne). 2025 Jan 30;11:1473862. doi: 10.3389/fmed.2024.1473862 (PMC11823639; doi:10.3389/fmed.2024.1473862)
Supplement: Supplementary file 1 [file Data_Sheet_1.docx]

Supplementary Material

# S1. Supplementary materials. Questionnaire for reference persons of off-patent paediatric drug development programs.

Dear Colleague,

We are contacting you as the coordinator of a research project funded by the European Commission's Seventh Framework Programme to conduct a paediatric development programme of off-patent medicines in paediatrics with a view to applying for a Paediatric Use Marketing Authorisation. We would like to ask you for some information in order to analyse the challenges related to the implementation of these development plans. This survey is part of a self-sustaining research initiative supported by the TEDDY European Network of Excellence for Paediatric Research (https://www.teddynetwork.net/).Most of the questions are multiple choice and should take no more than 10 minutes to complete. The data you provide will only be used for research purposes and will be published in a scientific paper as aggregated data. The data will be accessible only to the study team, which is bound by professional secrecy, and no personal data will be published. Fondazione per la Ricerca Farmacologica Gianni Benzi (Via Giulio Petroni, 91/B, 70124 Bari, Italy) is responsible for the processing of personal data. For more details, the Data Protection Policy is available here: https://www.benzifoundation.org/wp-content/uploads/2024/05/FGB_Dataprotectionpolicy_20052019_template2024.pdf

*Declaration*

*I have read previous information and I authorise the processing of personal data, in compliance with the General Data Protection Regulation 2016/679/EC for the specific purpose they are collected*

Yes

No

General information

Full name of the person filling in the questionnaire

Reference institution

Name

Please indicate the name of the project funded under the Seventh Framework Programme (FP7) in which you undertook paediatric development of one or more off-patent active substance*

Progress of the paediatric development program

Indicate the name of the active substance(s) you have studied in paediatric subjects

(Please refer to the Seventh Framework Programme funded project for which you are responding)

What is the status of the paediatric development program you undertook to study and develop the paediatric medicine?

(only one option applies)

Successfully terminated

Early terminated

Ongoing

Other, please specify

Please specify the status of the paediatric development program

At which stage did the paediatric development program end/stop?

(only one option applies)

Pharmaceutical development/manufacturing phase

Non-clinical phase (animal studies)

Clinical phase

After the completion of the R&D program, as required in the project for the MA

The paediatric development program is still ongoing

Other, please specify

Please specify at which stage the program ended/stopped

Did your paediatric development program involve a pharmaceutical company/SME that committed itself of applying for a Marking Authorisation of the paediatric medicine?

Yes

No

Other, please specify

Please specify the commitment of the pharmaceutical company/SME in the paediatric development program

Please indicate the main challenges (one to three) encountered by this paediatric development program

Responding/complying with Paediatric Committee (PDCO) requests

Development of age-appropriate drug formulations

Conduct of non-clinical studies

Responding/complying with national Competent Authorities and/or ethics committees requests

Long regulatory/ethics committee approval process for paediatric clinical trials

Challenges with drug supply

Safety/Efficacy issues

Recruitment challenges

Coordinating different stakeholders participating in the pediatric development program/Withdrawal of key stakeholders

Budgeting

Trial sites activation

Other, please specify

Please detail any other challenge not mentioned in the previous list

Please, comment on the main challenges you have mentioned

Did you agree a Paediatric Investigation Plan (PIP) with the PDCO?

Yes

No

If yes, did the PIP progress as planned?

Yes

No

If the PIP did not progress as planned, please explain why

Did you need to ask for PIP modifications?

Yes

No

What were the main aspects that required modifications to the PIP?

More than one answer is possible

Timelines

Sample size

Deletion of one or more clinical study(-ies)

Inclusion of one or more clinical study(-ies)

Study population

Dosage or dosing rules

Primary endpoint

Secondary endpoint(s)

Formulation

Non-clinical issues

Other, please specify*

Please, indicate any other aspect that required modifications to the PIP

Details on the Marketing Authorisation following the paediatric development program

The following questions apply only if you proceeded for a Marketing Authorisation covering paediatric subjects

In case of successfully terminated paediatric development program, did you apply for a Marketing Authorisation covering paediatric subjects?

Yes, we got a Paediatric Use Marketing Authorisation (PUMA)

Yes, we got a MA variation covering paediatric subjects

Application for a PUMA is ongoing

No, even if the paediatric development program was successfully terminated

No, the paediatric development program was not terminated

Other, please specify

Please specify if any other Marketing Authorisation pathway covering paediatric subjects is ongoing

In case you have completed the paediatric development program but you have not pursued for a Marketing Authorisation covering paediatric subjects, please explain why

Please specify which Marketing Authorisation Application procedure you followed

Centralised

National

Final remarks

Please, report any other hurdle/relevant aspect you have experienced in running pediatric drug development program for off-patent medicines under the Seventh Framework Programme

# Supplementary Table S1. Details of existing MAs for the active substances studied in the FP7-funded projects for development of off-patent medicines in paediatrics.

| **Active substance** | **Approved therapeutic indication(s)** | **Available pharmaceutical form(s)** | **Repurposing** | **Type of repurposing** | **Paediatric-only disease** |
| --- | --- | --- | --- | --- | --- |
| 6-mercaptopurine* | Acute lymphoblastic leukaemia in adults, adolescents and children | Tablets  Oral liquid formulation (authorized at centralized level when the FP7 funded consortium applied for a MA) | Yes | New pharmaceutical age-appropriate formulation/strength (oral liquid suspension) | No |
| Azithromycin* | Infections caused by azithromycin-sensitive germs  Prophylaxis of Mycobacterium avium-intracellulare infections in HIV patients  Bacterial conjunctivitis, in children (from birth to 17 years) and adults (eye drops)  Pneumonia/ inflammatory pelvic disease (solution for infusion) | Coated tablets  Eye drops  Powder for oral suspension, solution for infusion | Yes | New non-authorised indication (Prevention of bronchopulmonary dysplasia)  Extension to a new paediatric age subset (preterm/term neonates)  New pharmaceutical age-appropriate formulation/ strength (Age-appropriate dosage form for parenteral use) | Yes |
| Budesonide | Primary immunoglobulin A (IgA) nephropathy  Eosinophilic esophagitis  Asthma  Pseudocroup  BPCO  Dermopathies  Ichiness  Autoimmune hepatitis  Chron diseases  Ulcerative colitis  Allergic rhinitis  Nasal polyposis | Modified-release hard capsules Orodispersible tablets  Inhalation solution  Powder for inhalation  Ointment Enteric-coated hard capsules modified-release tablets or capsules  Nasal spray | Yes | New non-authorised indication (Prevention of bronchopulmonary dysplasia)  Extension to a new paediatric age subset (neonates) | Yes |
| Bumetanide | Renal and hepatic oedema  Heart failure, acute and chronic renal failure  In adults, hypertension with visceral damage threatening short-term vital prognosis | Tablets, solution for injection | Yes | New non-authorised indication (seizures in newborn babies with hypoxic ischemic encephalopathy)  Extension to a new paediatric age subset (preterm/term neonates)  New pharmaceutical age-appropriate formulation/ strength (Neonatal-appropriate dosage form) | Yes |
| Ciprofloxacin hydrochloride | Infections in adults  Management of neutropenic patients with fever due to bacterial infection  In children and adolescents, for bronchopulmonary infections caused by Pseudomonas aeruginosa  Serious infections in children and adolescents if deemed necessary | Coated tablets/ modified-release tablets  Granules and solvent for an oral suspension ear drops/ointment/suspension for ear instillation  Eye drops/eye ointment  Solution for infusion  Suspension for injection | Yes | Extension to a new paediatric age subset (preterm/term neonates)  New pharmaceutical age-appropriate formulation/ strength (Neonatal-appropriate dosage form) | No |
| Clonidine (hydrochloride) | Arterial hypertension  Hypertensive crises and hypertension where oral administration is not possible or ineffective (parenteral forms)  Ocular hypertension and glaucoma (eye drops) | Coated tablet  Hard capsule, modified-release  Transdermal patch  Solution for injection  Eye drops | Yes | New non-authorised indication (Sedation in intensive care)  Extension to a new paediatric age subset (from 0 to 18 years)  New pharmaceutical age-appropriate formulation/ strength (Different strengths vials for intravenous infusion) | No |
| Cyclophosphamide | Acute lymphoblastic leukemia, chronic lymphocytic leukemia  Lymphomas (Hodgkin's lymphoma, non-Hodgkin's lymphoma, multiple myeloma)  Ovarian and breast cancer  Ewing's sarcoma  Small-cell lung cancer  Metastatic or advanced tumors of the central nervous system (neuroblastoma)  Autoimmune Diseases: severe progressive lupus nephritis, Wegener’s granulomatosis  Breast cancer therapy | Powder for the preparation of a solution for injection/infusion  Tablet | Yes | Extension to a new paediatric age subset (from 0 to 18 years)  New pharmaceutical age-appropriate formulation/ strength (Soluble tablets) | No |
| Deferiprone* | Treatment of iron overload in patients with thalassaemia major when current chelation therapy is contraindicated or inadequate | Film-coated tablet and  Oral solution | Yes | Wider therapeutic indication (Treatment of iron overload in paediatric patients affected by haemoglobinopathies requiring chronic transfusion)  Extension to a new paediatric age subset (from 1 month to 18 years)  New pharmaceutical age-appropriate formulation/ strength (Oral liquid formulation) | No |
| Dobutamine (hydrochloride) | Indicated in adults requiring inotropic support for low output heart failure  Cardiac stress testing as an alternative to exercise in patients for whom routine exercise cannot be performed satisfactorily  Low-flow syndromes of different aetiologies  In all paediatric age groups (newborn to 18 years) for its positive inotropic effect in states of hypoperfusion due to low cardiac output | Solution for infusion | Yes | New non-authorised indication (Treatment of neonatal circulatory failure in the first 72 hours after birth)  New pharmaceutical age-appropriate formulation/ strength (Solution for injection with neonatal- appropriate excipients, volume, concentration and strength) | No |
| Dopamine | Low-flow syndromes of different aetiologies  Shock conditions eg in the case of heart failure, severe infections, hypersensitivity reactions, severe hypotension, acute renal failure | Solution for infusion/injection | Yes | Extension to a new paediatric age subset  New pharmaceutical age-appropriate formulation/strength (intravenous solution with different strengths) | No |
| Doxorubicin* | Different types of cancer in adults and children | Powder, dispersion and solvent for concentrate for dispersion for infusion | No | - | No |
| Enalapril (maleate) | Hypertension  Symptomatic heart failure treatment and prevention | Tablets  Solution for injection | Yes | Extension to a new paediatric age subset  New pharmaceutical age-appropriate formulation/strength (solid drug formulation for use in neonates and children) | No |
| Ethosuximide | Pyknoleptic absences  Complex and atypical absences  Myoclonic-astatic petit mal  Myoclonic seizures in adolescents | Tablets Prolonged released tablets  Soft capsules Hard capsules Syrup Solution Solution for injection | Yes | New pharmaceutical age-appropriate formulation/strength (granules) | No |
| Fentanyl (citrate) | Breakthrough pain in chronic cancer pain | Sublingual tablets Orosoluble tablets Nasal spray Transdermal patch Injectable solution | Yes | Wider therapeutic indication (Prevention of acute pain, treatment of acute pain, pre-medication before a painful medical procedure)  Extension to a new paediatric age subset | No |
| Fluconazole | In adults:  Fungal infections  Prophylaxis in patients at high risk to prevent relapse of: cryptococcal meningitis, oropharyngeal or esophageal candidiasis in HIV-infected patients, recurrent vaginal candidiasis  Prophylaxis of candidiasis in patients with prolonged neutropenia  In infants, children, and adolescents (0 to 17 years):  Mucosal candidiasis  Invasive candidiasis  Treatment /maintenance therapy to prevent relapse of cryptococcal meningitis  Prophylaxis of candidiasis in immunocompromised patients | Powder for oral suspension  Hard capsules  Solution for infusion | Yes | Extension to a new age group (preterm newborns) | No |
| Gabapentin | In combination for partial epilepsy with or without secondary generalisation in adults and children from 6 years of age  As monotherapy for partial epilepsy with or without secondary generalisation in adults and adolescents from 12 years of age  Peripheral neuropathic pain in adults | Capsules Coated tablets Oral solution | Yes | Extension to a new paediatric age subset  New pharmaceutical age-appropriate formulation/strength (oral solution) | No |
| Hydrocortisone* | Congenital adrenal hyperplasia  Adrenal insufficiency  Inflammation and allergic diseases of the anterior tract of the ocular globe.  Insect bites, itching, erythema or circumscribed burns, eczema  Psoriasis  Internal or external hemorrhoids  Eye/ear acute bacterial inflammation | Modified-release hard capsules/tablets Eye drops Topical/rectal cream Solution for infusion Injectable solution Eye ointment, ear ointment | Yes | Extension to a new paediatric age subset  New pharmaceutical age-appropriate formulation/strength (granules in capsules for opening) | No |

| Meropenem | Infections in adults and children over 3 months of age  Management of neutropenic patients with fever, bacteraemia in association with, any of the infections listed above | Powder for solution for injection / infusion | Yes | Extension to a new paediatric age subset (preterm/term newborns, infants aged < 3 months) | No |
| --- | --- | --- | --- | --- | --- |

| Metformin | Type 2 diabetes mellitus (as monotherapy or in combination with insulin), in adults and in children/adolescents from 10 years of age | Film-coated tablets, prolonged release tablets, dispersible tablets, effervescent tablets, powder for oral solution, oral solution | Yes | New non-authorised indication (Treatment of polycystic ovary syndrome as adjunct to diet and exercise in adolescent girls to improve menstrual regularity and insulin resistance)  Extension to a new paediatric age subset (adolescents)  New pharmaceutical age-appropriate formulation/strength (effervescent granules for oral use) | No |
| --- | --- | --- | --- | --- | --- |
| Methotrexate | Rheumatoid arthritis in adults  Psoriatic arthritis in adults  Polyarticular juvenile rheumatoid arthritis in children  Rheumatoid arthritis  Psoriatic arthritis  Polyarticular juvenile rheumatoid arthritis in children  Acute leukaemia (especially childhood leukaemia)  Choriocarcinoma, destructive chorioadenoma and vesicular or hydatidiform mola  Major solid tumours (sarcomas, lymphomas, cervico-facial carcinomas, carcinomas of the Breast, lung and cervix carcinomas)  Lymphoblastic leukaemia in children  Lymphosarcoma in children | Tablets  Solution for injection (also in pre-filled syringes)  Solution for infusion  Oral solution | Yes | New pharmaceutical age-appropriate formulation/strength (oral liquid suspension) | No |
| Morphine | Severe and very severe pain,  It is indicated in adults and children/Intense and/or intractable pain requiring continuous administration of morphine | Solution for injection/infusion, tablets and prolonged release tablets, oral solution | Yes | Extension to a new paediatric age subset (neonates)  New pharmaceutical age-appropriate formulation/strength (Intravenous formulation for neonatal use) | No |
| Risperidone | Schizophrenia, maniac episodes associated with bipolar disorders, persistent aggression in Alzheimer patients and in children < 5 yrs with conduct disorders | Film-coated tablets oral solution  Orodispersible tablets  Orodispersible films  Powder and solvent for prolonged-release suspension for injection | No | NA | No |
| Temozolomide* | Newly-diagnosed glioblastoma multiforme - with malignant glioma, such as glioblastoma multiforme or anaplastic astrocytoma in children from the age of three years, adolescents and adult patients | Hard capsules | Yes | New pharmaceutical age-appropriate formulation/strength (Powder for oral suspension) | No |
| Vancomycin | Complicated skin and soft tissue infections  Infections of the bones and joints  Community-acquired pneumonia  Infective endocarditis  Bacteraemia  Central nervous system infections in all age groups  Perioperative antibacterial Prophylaxis in patients at high risk of bacterial endocarditis  Infections caused by the bacterium Clostridium difficile in patients from 12 years on | Powder for solution for infusion/ injectable solution  Hard capsules  Powder for oral solution | Yes | Extension to a new paediatric age subset (preterm/term newborns, infants aged < 3 months)  New pharmaceutical age-appropriate formulation/strength (125mg powder for concentrate for solution for infusion for pre-term and neonates) | No |
| *Active substance having an ODD for the same condition studied in the FP7 paediatric development plan | | | | | |
